# Protocol for the analysis of a natural experiment on the impact of the Affordable Care Act on diabetes care in community health centers

**DOI:** 10.1186/s13012-017-0543-6

**Published:** 2017-02-10

**Authors:** Nathalie Huguet, Heather Angier, Miguel Marino, K. John McConnell, Megan J. Hoopes, Jean P. O’Malley, Lewis A. Raynor, Sonja Likumahuwa-Ackman, Heather Holderness, Jennifer E. DeVoe

**Affiliations:** 1 Department of Family Medicine, Oregon Health & Science University, 3181 SW Sam Jackson Park Rd, Portland, OR 97239 USA; 2 Department of Emergency Medicine, Oregon Health & Science University, 3181 SW Sam Jackson Park Rd, Portland, OR 97239 USA; 3 Research Department, OCHIN Inc., 1881 SW Naito Pkwy, Portland, OR 97201 USA

**Keywords:** Affordable Care Act, Diabetes, Natural experiment, Community health center

## Abstract

**Background:**

It is hypothesized that Affordable Care Act (ACA) Medicaid expansions could substantially improve access to health insurance and healthcare services for patients at risk for diabetes mellitus (DM), with pre-DM, or already diagnosed with DM. The ACA called for every state to expand Medicaid coverage by 2014. In a 2012 legal challenge, the US Supreme Court ruled that states were not required to implement Medicaid expansions. This 'natural experiment' presents a unique opportunity to learn whether and to what extent Medicaid expansion can affect healthcare access and services for patients with DM risk, pre-DM, or DM.

**Methods/design:**

Data from electronic health records (EHRs) from the Accelerating Data Value Across a National Community Health Center Network (ADVANCE) clinical data research network, which has data from >700 community health centers (CHCs), was included in the study. EHR data will be linked to Oregon Medicaid claims data. Data collection will include information on changes in health insurance, service receipt, and health outcomes, spanning 9 years (pre- and post-expansion), comparing states that expanded Medicaid, and those that did not. Patients included in this study will be diagnosed with DM, be at risk for DM, or have pre-DM, between the ages of 19 and 64, with ≥1 ambulatory visit. Sample size is estimated to be roughly 275,000 patients. Biostatistical analyses will include the difference-in-differences (DID) methodology and a generalized linear mixed model. Econometric analyses will include a DID two-part method to calculate the difference in Medicaid expenditures in Oregon among newly insured CHC patients.

**Discussion:**

Findings will have national relevance on DM health services and outcomes and will be shared through national conferences and publications. The findings will provide information needed to impact the policy as it is related to access to health insurance and receipt of healthcare among a vulnerable population.

**Trial registration:**

This project is registered with ClinicalTrials.gov (NCT02685384). Registered 18 May 2016.

## Background

Diabetes mellitus (DM) is one of the nation’s leading causes of morbidity and mortality [1, 2]. In 2012, >29 million people in the USA had DM, of which 1.7 million were newly diagnosed [3]. Uninsured patients are more likely to have undiagnosed DM, and the longer they lack insurance, the higher this likelihood [4]. Uninsured patients with DM are also less likely to receive recommended DM care and have poorer DM control than insured patients [5–8]; lack of health insurance can greatly exacerbate the challenges of successful DM care and management [9–13]. Previous research showed uninsured patients had lower odds of receiving DM-related services, even when they came in for a clinic visit, compared to insured patients at a similar visit [5]. Thus, health insurance and continued access to healthcare services are essential for optimal DM detection, care, and management.

It is hypothesized that Affordable Care Act (ACA) Medicaid expansions could substantially improve access to health insurance and healthcare services for patients at risk for DM, with pre-DM, or DM. The ACA is the largest healthcare-related legislation in the USA since Medicare’s establishment in 1966. With the goal of covering all low-income citizens and legal residents [14], the ACA called for Medicaid expansions to all individuals earning ≤138% of the federal poverty level (FPL). However, in 2012, the US Supreme Court ruled that states were not legally required to implement the Medicaid expansions [15]. As of April 2016, 32 states and the District of Columbia implemented expansions while 18 states did not [16]; current estimates show that Medicaid enrollment grew by 18% in expansion states and by 5% in non-expansion states [17]. This ‘natural experiment’ presents a unique opportunity to learn whether and to what extent Medicaid expansion can affect healthcare access and services for patients at risk for DM, with pre-DM, or DM. Here, we present the project aims, methods, and planned analyses for the project.

## Methods

### Study aims

To assess this natural policy experiment, we will use electronic health record (EHR) data from community health centers (CHCs) in states that expanded and did not expand Medicaid. The study has the following specific aims.

### Aim 1

Compare pre-post ACA insurance status, overall visits, and chronic disease management visits among patients with DM risk, pre-DM, or DM, among CHC patients in expansion versus non-expansion states.

### Aim 2

Compare pre-post ACA receipt of primary and secondary DM preventive services [e.g., screening for obesity, lipid levels, glycosylated hemoglobin (HbA1c)] among patients with DM risk, pre-DM, or DM, among CHC patients in expansion versus non-expansion states.

### Aim 3

Compare pre-post ACA changes in DM-related biomarkers (e.g., body mass index, blood pressure, lipid levels) in CHC patients with DM risk, pre-DM, or DM among newly insured (gained Medicaid in post-period), already insured (had Medicaid coverage in pre- and post-periods), and continuously uninsured (pre- and post-periods) patients in states that expanded Medicaid.

### Aim 4

Measure pre-post ACA changes in Oregon Medicaid expenditures among newly insured compared to already insured CHC patients with DM risk, pre-DM, or DM.

### Data sources

We will use EHR data from the Accelerating Data Value Across a National Community Health Center Network (ADVANCE) clinical data research network (CDRN) of PCORNnet (National Patient-Centered Clinical Research Network) [18]. The ADVANCE CDRN is a unique ‘community laboratory’ for research with underrepresented populations receiving care in CHCs—our nation’s safety net [18]. Led by the OCHIN (not an acronym) community health information network, the ADVANCE CDRN’s research-ready data warehouse integrates longitudinal outpatient EHR data from OCHIN, Health Choice Network (HCN), and Fenway Health. These three CHC networks primarily serve vulnerable populations and, as of June 2016, include 2,195 CHCs with >3.1 million active patients in 23 states (1194 CHCs in 12 states that implemented Medicaid expansion in January 2014, and 1001 CHCs in 11 states that did not). Of note, the project will use a more restricted database due to eligibility criteria as described below.

OCHIN is a collaborative that includes >450 CHCs and other community-based clinics [19]. OCHIN is the nation’s largest CHC network utilizing a single instance of one EHR system (and the only one using Epic©). Pioneering the implementation of a single, hosted instance of Epic© Systems EHR across hundreds of clinics, OCHIN maintains one enterprise-wide master patient index. Thus, OCHIN patients have a single medical record across all clinics in the network, and all data are managed centrally.

**HCN** has a history and organizational structure similar to OCHIN’s. In 1994, HCN was founded in Florida by a group of CHCs collaborating to recover from the impact of Hurricane Andrew; membership now spans 9 states. HCN members are hosted on a centralized EHR platform (Intergy™ by Vitera™) and supported by network-wide clinical informatics and analytic tools. In 2011, HCN partnered with OCHIN to develop the ADVANCE data and aggregation system. In addition to the ADVANCE research-ready data warehouse, this partnership has helped CHCs aggregate data for quality reporting, EHR “meaningful use,” and patient-centered medical home recognition.

Fenway Health, a free-standing CHC, was founded in 1971. In its early response to the AIDS epidemic, Fenway Health developed the capacity to support clinical research and has received significant federal funding [20]. Fenway has been a partner with OCHIN since 2010 [21]. Fenway has received national recognition for reducing healthcare disparities for sexual and gender minority populations, and is the home of the National Center for Lesbian, Gay, Bisexual and Transgender Health Education [22]. Fenway Health has had an EHR for >15 years and has participated in several national research consortia using EHR-based data.

We will also link OCHIN EHR data to Oregon Medicaid claims data in order to measure changes in Medicaid expenditures. Oregon’s Medicaid recipients are assigned unique individual identification (ID) numbers, facilitating data linkages across multiple databases, including the ADVANCE data warehouse. As we have done previously [23–25], we will use claims data from Oregon’s Medicaid Management Information System, recognized for exemplary data validation protocols by Centers for Medicare and Medicaid Services.

### Eligibility criteria

We will include patients with DM risk, pre-DM, or diagnosed DM, between the ages of 19 and 64, with one or more ambulatory visit. Data will derive from >700 CHCs in 20 states for which their EHR were ‘live’ as of 1/1/2013. We set these age criteria because the Medicaid expansion was aimed at adults aged 19 and older, many states’ Medicaid programs cover children through age 18, and nationally, individuals aged 65 and older are eligible for Medicare. We will exclude pregnant women to eliminate the possibility of having patients with gestational DM in the dataset.

### Definitions of patients with pre-DM, at-risk for DM, and DM

*Patients at-risk for DM*:

Patients aged 45 and older and with a BMI ≥ 25, following criteria from the Centers for Disease Control and Prevention [26].

*Patients with pre-DM*:

Patients with a single HbA1c between 5.7 and 6.4% and/or a fasting glucose between 100 and125 mg/deciliter.

*Patients with DM*:

The ADVANCE CDRN has developed a computable phenotype with criteria for identifying potential DM cohort members. This phenotype includes relevant information for identifying type 1 and type 2 DM. Both types of DM will be included in the study, although most patients have type 2 DM. Previous studies have validated the use of a similar method [27–29]. Patients with DM are identified as those with any combination of two “events” from outpatient diagnoses, diagnostic level laboratory results, or order of anti-hyperglycemic agents no more than 730 days apart. Examples:

1. At least two visits with a DM-related International Classification of Disease (ICD)-9 or 10 code,
2. One ICD-9/10-coded visit and one HbA1c or glucose test positive for DM, according to American Diabetes Association thresholds [30],
3. One ICD-9/10 coded visit and a diabetes-related medication order, or
4. A diabetes-related medication order and a positive HbA1c or glucose test.

Because some patients may not have the opportunity to have two events in the post-period (or pre-period), we risk an underestimation of patients with DM. Thus, we will conduct sensitivity analyses using one event to define DM patients and ensure robust findings. Table 1 shows a breakdown of ADVANCE patients with DM risk, pre-DM, or diagnosed with DM.

**Table 1:** ADVANCE patients (aged 19–64) with DM risk, pre-DM, or DM during the pre-period (01/01/2012-12/31/2013) by expansion status

	Total (*N* = 260,306)	Expansion^a^ (*N* = 141,353)	Non-expansion^b^ (*N* = 118,953)
DM categories^c^	*N* (%)	*N* (%)	*N* (%)
DM risk	169,045 (64.9)	91,607 (64.8)	77,438 (65.1)
Pre-DM	12,012 (4.6)	6991 (4.9)	5021 (4.2)
DM	79,249 (30.4)	42,755 (30.2)	36,494 (30.7)

^a^States who expanded Medicaid as of January 1, 2014 (CA, HI, MA, MD, MN, NM, NV, OH, OR, RI, and WA)

^b^States who had not expanded Medicaid as of January 1, 2014 (AK, IN, FL, KS, MO, MT, NC, TX, WI)

^c^DM categories defined prior to January 1, 2014

### Measures

This project has two main independent variables: Medicaid expansion status (states that expanded versus not) or insurance status.

#### Medicaid expansion status

**Fig. 1:**
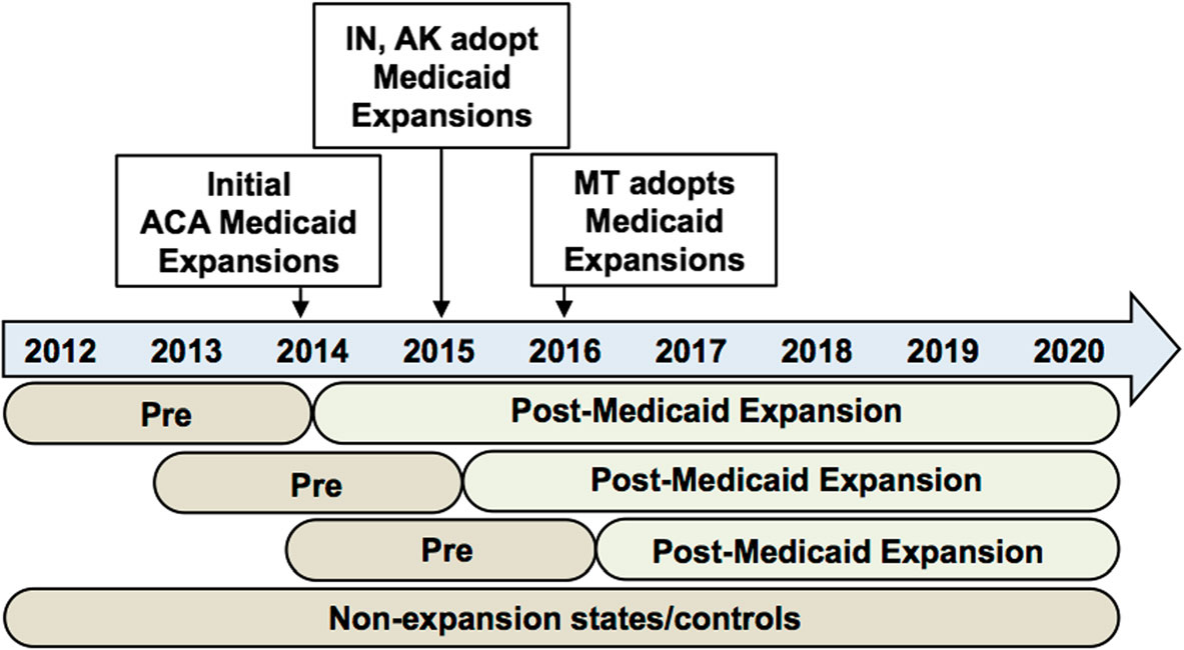
Medicaid expansion status timeline

We will define the pre- and post-Medicaid expansion periods based on if and when a state expanded Medicaid. As illustrated in Fig. 1, each row below the time arrow represents the pre-post periods depending on when ACA-sponsored Medicaid expansion was/will be implemented in states. Our study period will range from January 1, 2012 to December 31, 2020. We will have data spanning 9 years, which will allow us to examine the short-term (1–2 years) and medium term (4–5 years) impact of the Medicaid expansion on reducing disparities in access to and receipt of DM screening, treatment, and health outcomes as well as changes in expenditures. When the ACA-Medicaid expansion took effect on January 1, 2014, 14 states in the ADVANCE CDRN adopted Medicaid expansion. Their pre-period (first row) is thus 24 months prior to 2014 (i.e., 2012 through 2014) and their post-period is 2014 through 2020 (see first row in Fig. 1). Other states adopted the expansion later, such as Indiana and Alaska (pre/post period in row 2), Montana (pre/post period in row 3), and others (i.e*.,* Oklahoma) may change their policies during the remainder of the study period.

#### Insurance status

Since EHR health insurance data is primarily based on information collected at each visit [31], we propose to define newly insured, already insured, and continuously uninsured patients as follows:

1. Newly insured patients will have been uninsured at all visits in the pre-period and had all visits in the post-period paid by Medicaid;
2. Already insured patients will have all visits paid by Medicaid in both the pre- and post-periods;
3. Uninsured patients will have no coverage for all visits in both the pre- and post-periods.

Covariates will include sociodemographic variables (age, gender, race, ethnicity, poverty level, language preference, and urbanicity) and the frequency of healthcare visits.

#### Outcome measures

*Healthcare coverage* refers to patient’s health insurance status including coverage status, type of health insurance (e.g., Medicaid, private, Medicare) and percent of insured visits.

*Healthcare delivery* includes rates of all billed encounters (all, primary care visits, and mental and behavioral health encounters) and receipt of recommended preventive services [32, 33] (e.g., tobacco assessment, vaccinations, cholesterol screening, diabetic preventive care, blood pressure measurement, obesity screening, foot and retina exams, appropriate prescriptions).

We will also evaluate change in *DM-related biomarkers*, by identifying patients with elevated HbA1c (HbA1c >7% [34]), low-density lipoprotein (LDL *≥* 100 mg/dl [32–34]), blood pressure (last measure >140/90 mmHg), body mass index (BMI ≥ 30) and diabetes complications (e.g*.*, retinopathy, nephropathy, neuropathy). We will examine absolute changes in these biomarker values, as well as the proportion achieving control and rates of change.

*Medicaid expenditures* for services internal and external (Medicaid recipients only) to the patients’ clinic will be determined. Evaluating expenditures requires two steps.

1. For the subset of our cohort residing in Oregon, link patients with DM risk, pre-DM, or DM to Oregon’s Medicaid claims data.
2. Attach expenditures to uninsured individuals for services. Although these individuals do not have paid Oregon Medicaid claims, they do have encounter data that are captured by the ADVANCE data. These encounter data include the Current Procedural Terminology (CPT) codes that identify the services rendered to those patients. We will attach an expenditure for each service based on its average Medicaid fee-for-service reimbursement in the first year of the sample. In other words, a standardized price will be defined for each service provided by the clinic. In order to be consistent, we will attach these prices to all patients, including those with insurance. These repriced claims reflect differences in utilization only (and not payment or capitation rates). The investigators have used this approach previously [35].

Additional outcome variables may be added if more data become available.

### Analytic procedures

#### Biostatistical analyses

Our primary methodological approach to address study aims will utilize difference-in-differences (DID) methodology [36–38]. To address aims 1–3, we will use a generalized linear mixed model (GLMM) approach to adjust for serial correlation and other potential confounders. An interaction term for Medicaid expansion (or insurance status) and a post-expansion indicator variable will be included in the model to determine the DID in the outcomes. Additionally, this model is flexible enough to construct models stratified by patients with DM risk, pre-DM, or DM. Further, we will stratify the models by types of insurance (i.e., Medicaid vs. private/employer-sponsored) because healthcare delivery may vary by type.

Moreover, we will test three-way interaction terms of demographic indicators (i.e., race/ethnicity, gender, age), time, and Medicaid expansion indicator. We will compare the potential effect of gaining insurance on DM, pre-DM, or DM risk patients’ care, outcomes, and expenditures by demographic characteristics because of the differential prevalence of DM between these sociodemographic groups.

We will use propensity score weighting methods to reduce the observed bias, help minimize external threats to the validity of the results, and adjust for imbalances between expansion and non-expansion groups [39]. Clinic and patient panel characteristics that remain unbalanced between the intervention and control groups after propensity score adjustment will be included as covariates in the GLMM models to control for residual confounding. Longitudinal GLMM models will account for correlation within matched clinic site pairs and within CHCs through random effects.

#### Econometric analyses

To address study aim 4, using DID methods, we will calculate the average pre-post difference in Medicaid expenditures at the CHC attributable to the subpopulation of Oregon newly insured DM risk, pre-DM, or DM patients in ADVANCE data, subtracted by the average difference among the already insured patients. We will use a well-validated approach for modeling this phenomenon: the 2-part model [40]. Part 1 will use logistic regression to estimate the probability of any expenditure. Part 2 will focus on individuals with non-zero expenditures. We will use recent literature to guide the appropriate estimation approach, taking into account the potentially skewed distribution of the dependent variable [41, 42].

This study was reviewed and approved by the Oregon Health & Science University Institutional Review Board. It is registered with ClinicalTrials.gov (NCT02685384).

## Discussion

By making the Medicaid expansion optional for states, the US Supreme Court created a natural policy experiment to analyze the impact of a large-scale, national expansion of Medicaid on DM prevention and treatment. Our study capitalizes on this natural experiment by including data from 20 states; analyses will uniquely inform national and state policy decisions as states grapple with how to equitably distribute healthcare resources after the passage of federal health reform. To be useful, health policy reform evaluation must be timely, yet data from most currently available national sources have several years’ delay between data collection and analysis. EHR data overcome these limitations as they are current and can provide information about the immediate, real-time impacts of the ACA’s policies on clinic populations.

Fortunately, the rapid growth of EHR use in CHCs serving vulnerable populations yields unprecedented opportunities for real-time evaluation of how health policy changes impact access to care, and utilization and delivery of CHC services. Further, because CHC patients are primarily low-income, racial/ethnic minorities, and/or from rural populations, this study will monitor and evaluate whether the ACA Medicaid expansion mitigated health disparities, especially among patients with DM.

This study has some limitations. First, EHR data are not originally developed for research; however, we have conducted multiple data validation studies, built many EHR research datasets, and successfully conducted policy-relevant research using EHR datasets in the past [24, 43, 44]. Second, we anticipate missing data, either from services documented inaccessibly in the EHR (likely random) or from patients who went outside the ADVANCE CDRN to receive services (perhaps not random). Our analyses can accommodate missing data resulting from patient attrition. We will model missingness by including related variables in the analysis as covariates [45] or using a method such as multiple imputation to include these patients in analyses [46]. Third, as with any ‘real-world’ study, unobserved changes may occur over time, making it difficult to isolate the effect of the ACA.

In conclusion, this project assesses the natural policy experiment created by ACA Medicaid expansions when some US states expanded Medicaid while others did not. Findings will have national relevance on DM prevention, diagnosis, treatment, expenditures, and health outcomes. It investigates how Medicaid expansions impact access to and changes in receipt of healthcare services among a vulnerable population of patients with DM risk, pre-DM, or DM and creates validated data sources for studying vulnerable populations.

## Abbreviations

ACA: Affordable Care Act
ADVANCE: Accelerating Data Value Across a National Community Health Center Network
BMI: Body mass index
CDRN: Clinical data research network
CHC: Community health centers
CPT: Current Procedural Terminology
DID: Difference-in-differences
DM: Diabetes mellitus
EHR: Electronic health record
FPL: Federal poverty level
GLMM: Generalized linear mixed model
HbA1c: Glycosylated hemoglobin
HCN: Health Choice Network
ICD: International Classification of Disease
LDL: Low-density lipoprotein
